# Alpha-Gal Syndrome in a Pediatric Patient From Northeast Florida

**DOI:** 10.7759/cureus.104623

**Published:** 2026-03-03

**Authors:** Neha J Rahalkar, Madeline R Sanders, Sunil N Joshi, Steve M Dorman, Hary T Katz

**Affiliations:** 1 Allergy and Immunology, Family Allergy and Asthma Consultants, Jacksonville, USA; 2 Allergy and Immunology, University of Florida College of Medicine, Jacksonville, USA

**Keywords:** alpha-gal syndrome, clinical case report, food hypersensitivity, pediatric allergy, tick-bite

## Abstract

Alpha-gal syndrome (AGS) is a rare, tick-borne condition that is characterized by a delayed hypersensitivity reaction to galactose-α-1,3-galactose (alpha-gal), a carbohydrate found in mammalian meat products. AGS is increasingly recognized in adults; however, cases often remain underreported or misdiagnosed in children. We report on a 10-year-old male in Northeast Florida presenting with gastrointestinal symptoms, urticaria, and oropharyngeal itching. These symptoms developed hours after consuming a cheeseburger. His medical history revealed multiple previous Lone Star tick bites. After an alpha-gal IgE panel was performed, the elevated specific IgE levels, in addition to the patient’s prior history of tick bites, led to a diagnosis of AGS. The patient was prescribed avoidance of mammalian meat and an epinephrine auto-injector. There is a growing need for correct symptom recognition in pediatric cases, and improved clinical recognition may facilitate earlier diagnosis in pediatric patients.

Florida’s warm climate and abundant wooded and marshy areas support tick activity. Unlike typical IgE-presenting allergens, AGS reactions may occur hours after ingestion, leading to cases remaining underreported or misdiagnosed. Repeated exposures can lead to more severe symptoms, emphasizing the need for early diagnosis. With very limited surveillance, the true prevalence and incidence of AGS in Florida are unknown due to underreporting, and this case report sheds light on the clinical presentation of a pediatric AGS case in Northeast Florida.

## Introduction

Alpha-gal syndrome (AGS) is a tick-borne allergy to mammalian meat, which is characterized by a delayed hypersensitivity reaction to galactose-α-1,3-galactose (alpha-gal). The Lone Star tick bite, specifically, is identified as the primary vector of cases of AGS in the United States [1,2]. The development of IgE-mediated allergic reactions is typically associated with immediate symptom onset after antigen exposure. However, in the case of AGS, the reaction is delayed, often occurring more than four hours after the consumption of mammalian meat. Repeated exposure may lead to the development of increasingly severe allergic reactions, which makes early detection and diagnosis crucial [3]. These episodes are characterized by urticaria, pruritus, angioedema, and anaphylaxis, which can lead to significant morbidity and even mortality without immediate treatment. The delayed presentation of AGS leads to delayed diagnosis or misdiagnosis due to the variations in symptom presentation [4]. AGS is diagnosed using a lab panel of alpha-gal-specific IgE, and alpha-gal sensitization is indicated by antibody levels > 0.10 kUA/L [5]. This case report describes the presenting symptoms of AGS in a pediatric patient post exposure to mammalian meat following repeated Lone Star tick bites. A secondary form of this allergy is described in patients who have received intravenous infusions of cetuximab [6].

Case reports of AGS rarely describe the presentation in pediatric populations. Pediatric AGS patients are more likely to present with urticaria and gastrointestinal symptoms, including diarrhea, nausea, and vomiting. Adult presentations also commonly include gastrointestinal symptoms, but also more frequently include delayed, allergic reactions, including urticaria and anaphylaxis, as opposed to pediatric populations [3]. In Florida, tick season is perennial, and bites can occur at any point during the year, as opposed to other regions of the country. North Central Florida specifically is identified as the most habitable region in the state for ticks; however, the most recent data available on the incidence or prevalence of Lone Star tick bites in the state is almost a decade old (2017) [7-9]. In addition, as of 2025, the Florida Department of Health does not track cases of AGS [10]. While cases of AGS are more common in the Central United States, to our knowledge, there are no published case reports regarding pediatric AGS in Florida at the time of publication. This case report serves to add to the growing body of literature regarding pediatric AGS.

## Case presentation

We present a 10-year-old male from Northeast Florida who presented with generalized urticaria, itchy ears, itchy throat, and vomiting approximately six hours after consumption of a cheeseburger. The symptoms resolved after administration of diphenhydramine at home. He had no prior history of any known food allergies, wheezing, or other asthma-like symptoms. A few weeks later, approximately three to four hours after ingesting another cheeseburger, he developed nausea followed by urticaria and was treated again with diphenhydramine by his caregiver at home. It was noted that the patient had a history of prior Lone Star tick bites from the family's lake home in Central Florida. Differential diagnoses considered included spontaneous urticaria, other IgE-mediated food allergies, and mast cell activation syndrome. However, given the convincing history of delayed anaphylaxis associated with mammalian meat ingestion, an alpha-gal-specific IgE panel was ordered. The alpha-gal panel demonstrated an elevated specific IgE level (76.5 kUA/L) consistent with alpha-gal sensitization. The delayed onset of symptoms following mammalian meat ingestion, personal patient history of Lone Star tick bites, and elevated specific IgE level for alpha-gal confirmed the diagnosis of AGS. Given the strongly positive result, no other laboratory evaluations were clinically relevant. He was advised to completely avoid mammalian meat and was prescribed an epinephrine auto-injector. Clinical characteristics of the case are described in Table 1. The patient was advised to follow up yearly to retest the IgE level for alpha-gal. At follow-up, the patient continues to carry an epinephrine auto-injector and noted no recurrent reactions after avoidance of mammalian meat.

**Table 1:** Summarized clinical characteristics of the case Titer value (kUA/L) classification: Negative titer (less than 0.10 kUA/L), Borderline (0.10-0.34 kUA/L), Equivocal (0.35-0.69 kUA/L), Positive (0.70-17.4 kUA/L), Strongly positive (17.5 ≤). Reference values were sourced from [5].

Case Characteristic	Finding
Age (years)	10
Sex	Male
Type of Mammalian Meat Trigger	Beef
Region	Northeast Florida
History of Multiple Bites (Yes/No)	Yes
Alpha-gal Specific IgE Titer Value (kUA/L)	76.5 (Strongly Positive)
Time for Symptom Onset Following Exposure	4 to 6 hours
Presenting Symptoms	Generalized Urticaria, Itchy Ears, Itchy Throat, Vomiting
Treatment	Complete Avoidance of Mammalian Meat, Prescribed Epinephrine Auto-Injector
Outcome	No Recurrence of Symptoms After Avoidance

## Discussion

Search strategy

A narrative literature search was performed using the National Institutes of Health (NIH) database PubMed using the keyword “alpha-gal syndrome” and filtered by “human,” “children 0-17,” “case report,” and “English.” Additional data on incidence, prevalence, and clinical presentation were sourced from PubMed, the CDC, and the Florida Department of Health. This narrative review yielded nine articles, narrowed to five, prioritizing pediatric case reports. Exclusions included mixed-age studies that failed to detail specific symptom presentation of AGS in pediatric patients, cases linked to non-meat exposures (e.g., vaccination), and those involving children with pre-existing chronic conditions.

Narrative literature review

While larger pediatric cohort studies describing AGS have been reported, they do not provide individual patient-level detail as compared to case reports [11,12]. We reviewed five case reports detailing individual pediatric cases of AGS. Patient demographic information is detailed in Table 2. Of the reviewed case reports, four out of the five were male pediatric patients, and the average age was seven and a half [4,13-16]. It is worth noting that all cases were relatively consistent in age, occurring in young children (under 10). The reported mammalian meat trigger of the described patients was also reported more commonly as beef or pork when specified [4,13,16]. Similarly, our case of a 10-year-old male patient identified beef as his primary trigger. Table 3 summarizes the history of these five cases. Urticaria was present in all cases, while gastrointestinal symptoms were present in four [4,13,15,16]. In our case, however, the patient also reported throat and ear itching, a symptom not mentioned in other publications. In cases reporting time of symptom onset, the average was four-six hours after exposure [13-16], which is consistent with our case. Alpha-gal IgE levels were obtained in every case with a broad range (0.2 kUA/L to 49.6 kUA/L), with an average of 14.8 kUA/L. Alpha-gal IgE levels demonstrated no consistent relationship with more symptoms in this sample [13-16]. Our patient presented with a relatively high IgE value of 76.5 kUA/L and experienced multiple significant symptoms, including generalized urticaria, itchy ear, itchy throat, and vomiting. Existing pediatric cohort studies describing AGS also demonstrate both diverse symptoms and an unclear relationship between IgE levels and severity in symptoms [11,12,17]. Our limited number of reviewed cases makes our findings descriptive rather than conclusive. Research that examines severity in relation to AGS-specific IgE levels is warranted to establish if a correlation exists between specific IgE value and severity of symptoms.

**Table 2:** Baseline demographics of existing literature

Author	Kinoshita et al. (2019) [13]	Keles et al. (2019) [16]	Enders et al. (2023) [4]	Khoury et al. (2018) [14]	Banovic et al. (2025) [15]
Age (years)	6	7	7	8	10
Sex	Male	Male	Male	Male	Female
Type of Mammalian Meat Trigger					
Beef	X		X		
Pork	X		X		
Unspecified		X		X	X
Region	Missouri	Turkey	Unspecified	New York	Serbia

**Table 3:** Baseline characteristics of presenting pediatric symptoms in existing literature Titer value (kUA/L) classification: Negative titer (less than 0.10 kUA/L), Borderline (0.10-0.34 kUA/L), Equivocal (0.35-0.69 kUA/L), Positive (0.70-17.4 kUA/L), Strongly positive (17.5 ≤). Reference values were sourced from [5].

Author	Kinoshita et al. (2019) [13]	Keles et al. (2019) [16]	Enders et al. (2023) [4]	Khoury et al. (2018) [14]	Banovic et al. (2025) [15]
Type of Mammalian Meat	Beef/Pork	Beef	Beef/Pork	Unknown	Unknown
History of Multiple Bites (Yes/No)	Yes	Yes	Yes	Yes	Yes
Titer Value	0.35 kUA/L (Equivocal)	2.87 kUA/L (Positive)	21.2 kUA/L (Strongly Positive)	49.6 kUA/L (Strongly Positive)	0.2 kUA/L (Borderline)
Time for Symptom Onset Following Exposure					
4-6 hours	X	X			
Unknown			X	X	X
Presenting Symptoms					
Urticaria	X	X	X	X	X
Nausea	X	X			
Abdominal Pain	X				X
Diarrhea	X				
Vomiting			X		
Shortness of Breath			X		
Facial Swelling			X		

Geographic region

To our knowledge, no studies reporting cases of pediatric AGS in Florida were identified at the time of publication. The exact incidence and prevalence of AGS are uncertain in the state, with the most recent research being from almost a decade prior [18]. Florida’s warmer climate and high prevalence of wooded areas and marshes create an ideal environment for increased tick activity [9], and the Lone Star tick bite is reported to be the most common tick bite in Northeast Florida. Additionally, Centers for Disease Control and Prevention (CDC) data demonstrate that Northeast Florida had between 11 and 87 suspected cases of AGS from the year 2017 to 2022 [19]. The lack of pediatric case reports for AGS in Florida suggests that AGS may be mistakenly attributed to other diseases. The true burden is likely underestimated, as AGS is not nationally notifiable through the CDC, and surveillance of AGS largely depends on laboratory testing data as opposed to mandatory reporting [10]. Further research and awareness of AGS in Florida are crucial for a better understanding of its incidence in a state that has the ideal climate for a longer tick season.

Diagnostic barriers

Many pediatric AGS cases face diagnostic delays due to nonspecific symptoms like GI distress and urticaria, which are the more common manifestations in pediatric cases [3]. One study comprising both adults and children noted the average time from initial symptom presentation to AGS diagnosis as 7.1 years, if not diagnosed in the first year [20]. Enders et al. report on a seven-year-old male who was misdiagnosed with chronic spontaneous urticaria two years before his AGS diagnosis. Alpha-gal IgE revealed an elevated level of 21.2 kUA/L, which confirmed a diagnosis of AGS [4,5]. Our patient was also undiagnosed for approximately six weeks from symptom presentation, despite a history of Lone Star tick bites, and was seen in our practice after an episode of itchy throat, nausea, and urticaria following the consumption of mammalian meat.

## Conclusions

This case report underscores the importance of clinical awareness of pediatric AGS and the need to further educate healthcare providers on the differences in symptomatology of the disease in adults and children to facilitate a rapid and accurate diagnosis.

A comprehensive history, including a history of prior tick bites, food allergies, and symptom onset relative to mammalian meat exposure, is critical for a timely and accurate diagnosis. In children who live in tick-endemic areas and present with urticaria, angioedema, and/or gastrointestinal symptoms, the diagnostic work-up should include lab testing for alpha-gal IgE to assess for AGS. Clinicians and public health officials must prioritize awareness and epidemiological surveillance of AGS in Florida, where a paucity of data exists on its incidence and prevalence.
